# A procedure to characterize geographic distributions of rare disorders in cohorts

**DOI:** 10.1186/1476-072X-7-26

**Published:** 2008-05-28

**Authors:** Karla C Van Meter, Lasse E Christiansen, Irva Hertz-Picciotto, Rahman Azari, Tim E Carpenter

**Affiliations:** 1 Department of Public Health Sciences, School of Medicine, University of California, Davis, USA; 2 Center for Animal Disease Modeling and Surveillance, University of California, Davis, USA; 3 Informatics and Mathematical Modelling, Technical University of Denmark, Lyngby, Denmark; 4 Center for Children's Environmental Health, University of California, Davis, USA; 5 Department of Statistics, University of California, Davis, USA; 6 Department of Medicine and Epidemiology, School of Veterinary Medicine, University of California, Davis, USA

## Abstract

**Background:**

Individual point data can be analyzed against an entire cohort instead of only sampled controls to accurately picture the geographic distribution of populations at risk for low prevalence diseases. Analyzed as individual points, many smaller clusters with high relative risks (RR) and low empirical p values are indistinguishable from a random distribution. When points are aggregated into areal units, small clusters may result in a larger cluster with a low RR or be lost if divided into pieces included in units of larger populations that show no increased prevalence. Previous simulation studies showed lowered validity of spatial scan tests for true clusters with low RR. Using simulations, this study explored the effects of low cluster RR and areal unit size on local area clustering test (LACT) results, proposing a procedure to improve accuracy of cohort spatial analysis for rare events.

**Results:**

Our simulations demonstrated the relationship of true RR to observed RR and p values with various, randomly located, cluster shapes, areal unit sizes and scanning window shapes in a diverse population distribution. Clusters with RR < 1.7 had elevated observed RRs and high p values.

We propose a cluster identification procedure that applies parallel multiple LACTs, one on point data and three on two distinct sets of areal units created with varying population parameters that minimize the range of population sizes among units. By accepting only clusters identified by all LACTs, having a minimum population size, a minimum relative risk and a maximum p value, this procedure improves the specificity achieved by any one of these tests alone on a cohort study of low prevalence data while retaining sensitivity for small clusters. The procedure is demonstrated on two study regions, each with a five-year cohort of births and cases of a rare developmental disorder.

**Conclusion:**

For truly exploratory research on a rare disorder, false positive clusters can cause costly diverted research efforts. By limiting false positives, this procedure identifies 'crude' clusters that can then be analyzed for known demographic risk factors to focus exploration for geographically-based environmental exposure on areas of otherwise unexplained raised incidence.

## Background

Spatial analysis is being used increasingly to generate hypotheses for non-infectious diseases. As such, it has an important role examining putative environmental risk factors for disorders with uncertain etiology. Two types of tests are commonly used to detect spatial clustering, local area clustering tests (LACTs) and global clustering tests. LACTs identify specific points or areas of clustering, while global clustering tests determine a generalized, non-specific pattern of clustering. Given the potential presence of these two cluster patterns, combined with the fact that spatial tests have relatively low power, it is recommended that the two types of tests be used in combination [1-5].

Simulation studies have further questioned the power of spatial cluster detection methods, especially when the cluster is irregularly shaped [6-10], located near borders or areas of low population density [2] or the elevated relative risk (RR = incidence proportion inside the cluster/incidence proportion outside the cluster) is small [2,8,11].

Further test validity issues arise when analyzing rare conditions, where false positive results are more common. Rare disorders require a large study population, which may be created either by aggregating cases over many years and/or using a large geographical area, to provide adequate cases for spatial analysis. (The possible confounding of time trends over a study period can be analyzed separately.) Spatial cohort data are now available for spatial analysis; however, many methods cannot analyze large datasets, necessitating the aggregation of point information into areal units. Problems associated with areal unit-based tests are that, in addition to a loss of information, a large population range across units can create artifactual patterns [12,13] and tests may fail to identify actual clusters at the extremes of that range [14].

In an attempt to minimize test inaccuracies, Jacquez et al [15] suggested using multiple spatial tests at different resolutions, looking for approximate agreement among different tests at different scales. SaTScan [16], a local area cluster test (LACT) and Maximized Excess Event Test (MEET) [14], a global clustering test, are among the most powerful and commonly used tests in their respective categories [3,6,8]; however, likely due to SaTScan's application of a fixed shape scanning window, it tends to overestimate the true cluster [5]. Two recently developed, flexibly-shaped LACTs, FleXScan [10] and Episcan [11], perform comparably to and are based on the same likelihood method as SaTScan.

The aim of this study was to evaluate a procedure, which combined LACTs with a global area test, in an effort to detect clustering of a rare disorder of unknown etiology. In addition, various scan window shapes, acceptance criteria and aggregation unit sizes were examined for alternative cluster shapes. Evaluation criteria were the procedure's sensitivity, specificity and probability of false positives. These results dictated additional criteria included in the procedure described for exploring rare disorders in cohort data.

## Results

### Simulations

#### Simulation set 1: Effect of areal unit population size

As with any test, the effectiveness of spatial tests for various data may be evaluated based on its sensitivity (SE = locations in true cluster identified as being in a cluster by a LACT/all locations in the true cluster), specificity (SP = locations not in the cluster that are identified as not being in the cluster by a LACT/all locations not in the true cluster) and whether there are strong influences due to a few cases.

In the first set of simulations, SE and SP were measured both for the actual cluster locations and for all areal units containing part of the cluster. As expected, the exact point-based median SE was higher than the areal unit-based median SE and the opposite was true for the median SP. All of the following results are point-based median SE and SP. Description of the simulation method follows in the Methods section.

**Figure 1:**
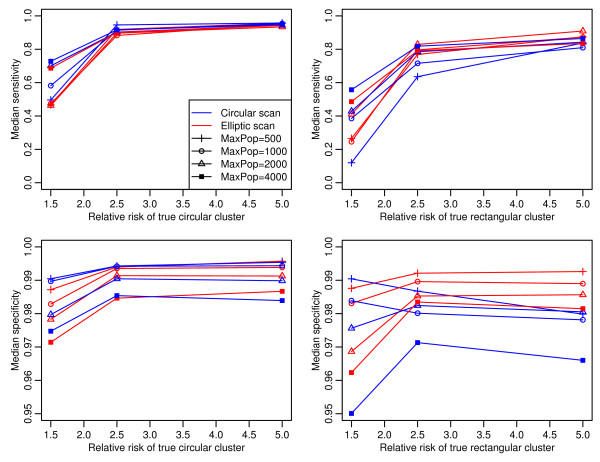
**Median sensitivity and specificity for varied areal unit population sizes and cluster relative risks. (Legend applies to all subplots.)**. A circular scan and elliptical scan were used to find the most likely cluster for each simulation. Two basic cluster shapes were used: circle and a rectangle with varying aspect ratio from 2 to 10. The cluster encompassed approximately five percent of the locations with a risk ratio of 1.5, 2.5 or 5.0. The study area was partitioned into areal units of maximum populations using the Epiunit method. Partitioning sets used had maximums of 500, 1,000, 2,000 or 4,000 locations per unit. Each scenario was simulated 100 times with a background prevalence of 0.004 and the location distribution of RC A as background.

Median SP and SE typically showed larger increases between RRs of 1.5 and 2.5, followed by little change between RRs of 2.5 and 5.0 for simulations of both the circular and rectangular cluster shapes using both circular and elliptical scanning windows (Figure 1). As n_max_, the population range parameter used to create the areal units, increased (500, 1000, 2000, 4000), median SE increased and median SP declined. For the rectangular cluster simulations with RR > 1.5, the elliptical scan median SP was a little higher than the circular scan's, though overall median SP is lower for this cluster shape. Median SP for the circular scan, using areal units with n_max _= 4,000 on rectangular clusters, was 0.1 below all others.

The largest increases for median SE with each doubling of n_max _were in the poor results at RR = 1.5. At RRs of 2.5 and 5.0, increases in median SE with increased n_max _are much smaller, especially in the circular cluster simulations. For the rectangular clusters, the median SE for n_max _= 500 (both scans below 0.30) was very low and the median SP for n_max _= 4,000 (both scans below 0.965) was low for a rare event.

#### Simulation set 2: Effect of RR

**Figure 2:**
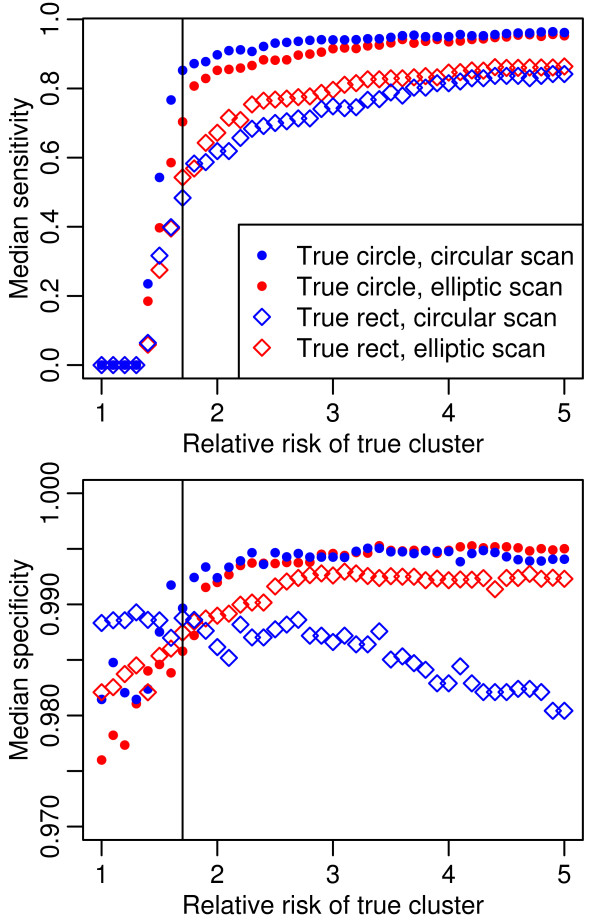
**Median sensitivity and specificity for true cluster relative risk varied from 1 to 5**. The vertical line is at a true relative risk of 1.7. As in Figure 1, two cluster shapes and two scanning window shapes were used for each simulation scenario. The simulation method was the same as for Figure 1, except that the maximum population per unit was held at 1,000 and the true relative risk was varied from 1 to 5 in 0.1 increments.

**Figure 3:**
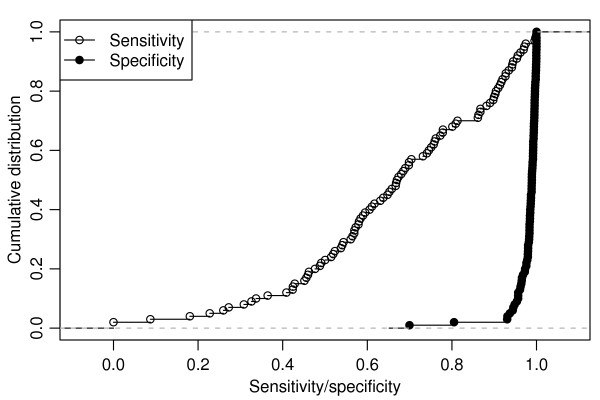
**Sensitivity and specificity for a simulated rectangular cluster using an elliptical scan**. One hundred simulations of a rectangular cluster with aspect ratio varying from 2 to 10, relative risk = 2, background prevalence = 0.004 and n_max_= 1,000. The higher aspect ratios create narrow elongated clusters that are harder to capture giving the tests lower sensitivity and having less effect on specificity.

Results from the second set of simulations, with n_max _fixed at 1,000, are summarized in Figures 2 through 5. Figure 2 shows the SE and SP for both scan shapes separately for each cluster shape. Figure 2 shows a dramatic improvement up to a true RR of 1.7 in SE for all scenarios. Improvement continues at a decreasing rate above that. SP also increases more up to RR of 1.7 for all but the circular scan on a rectangular cluster, but these differences are small. SE and SP are explored further in Figure 3, showing the cumulative distribution for the 100 simulations of the rectangular cluster with RR = 2.0, tested with an elliptical window.

**Figure 4:**
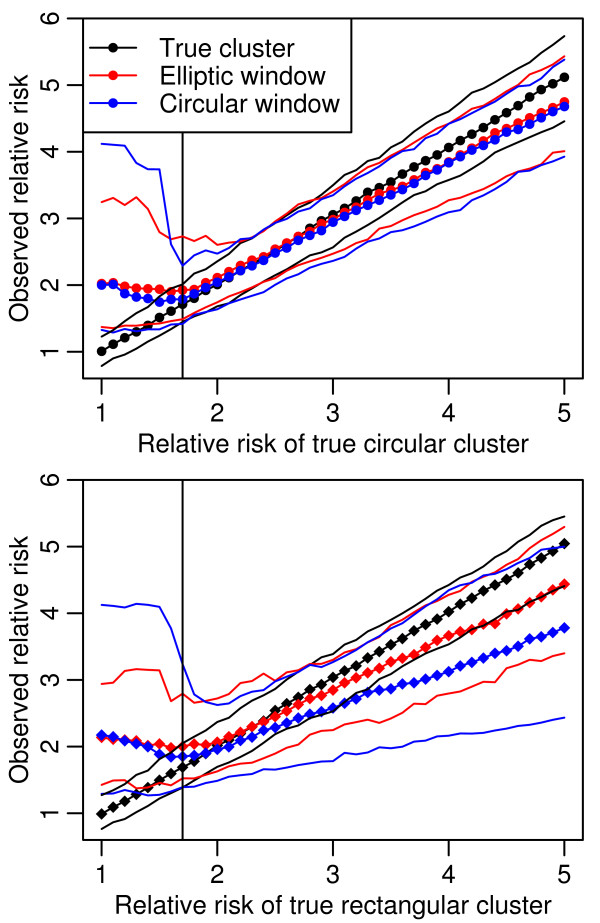
**Observed relative risks compared to the relative risk of the true simulated cluster**. For the Figure 2 simulations, dots represent median values and the solid lines are the 95 and 5 percentiles for each distribution. The true relative risk has a stochastic distribution as the actual number of locations in a cluster could vary a little from the specified risk when some locations were on the boundary of the defining circle or rectangle. As in Figure 1, there is a vertical line at a true relative risk of 1.7.

**Figure 5:**
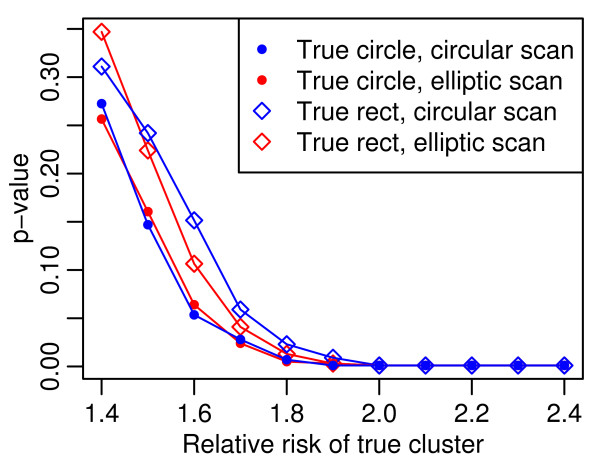
**Median p values for simulated clusters with varied relative risks**. For the Figure 2 simulations, the p values over a range of relative risks for the simulated circles and rectangles with varying aspect ratios and 1,000 maximum locations per areal unit, background prevalence of 0.004. Results for most likely cluster from circular and elliptical scan tests.

The important epidemiologic questions of the validity of the observed RR and p value are illustrated in Figure 4. For the circular cluster, the median observed RR converged to the true RR, when the latter was 1.7. For the rectangular cluster, convergence occurred at a true RR of 2.0, though it was near at 1.7. The cluster tests overestimated the RR when the true RR was low and underestimated when it was high. The magnitude of the overestimation at low true RR was not affected by true cluster shapes, though the circular scan overestimation was greater than that of the elliptical scan. When the true RR was above 2.5, the magnitude of the underestimation of the RR was similar and modest for both scan tests on circular clusters; however, when the clusters were rectangular, the underestimation increased, especially in the case of the circular scan test.

In Figure 5 median p values for the lower (< 1.7) true RRs reflect the wide range of observed RR shown in Figure 4. These results informed our spatial testing procedure and the criteria for a positive result, termed Consensus or Potential Clusters, which are described in the methods section.

## Illustration for test procedure

The mother's address at birth of the 1996 through 2000 State of California Department of Health Services Center for Health Statistics Confidential Birth Files [17] were geocoded. Cases diagnosed through February 2006 identified from the records of the California Department of Developmental Services (DDS), which assesses eligibility and coordinates services for people diagnosed with various developmental disorders, were matched to the birth records. The DDS divides California into twenty-one catchment regions, each serviced by an independent Regional Center (RC). Two of these were analyzed using the procedure presented in this paper. RC A, with mixed rural and urban areas, has one of the largest geocoded birth populations (219,417) in the state and a relatively low incidence, 28.6/10,000 births (628 cases). RC B serves a mostly urban area with 95,977 births geocoded and a relatively high overall incidence, 70.2/10,000 births (674 cases).

**Figure 6:**
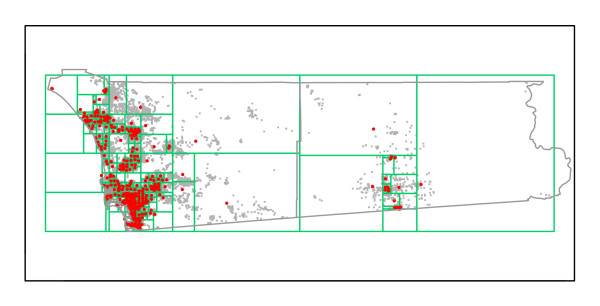
**Set 1 Epiunit areal units with maximum 1,000 births, for RC A**. Population centroids for each unit are red. All located births are gray points.

**Figure 7:**
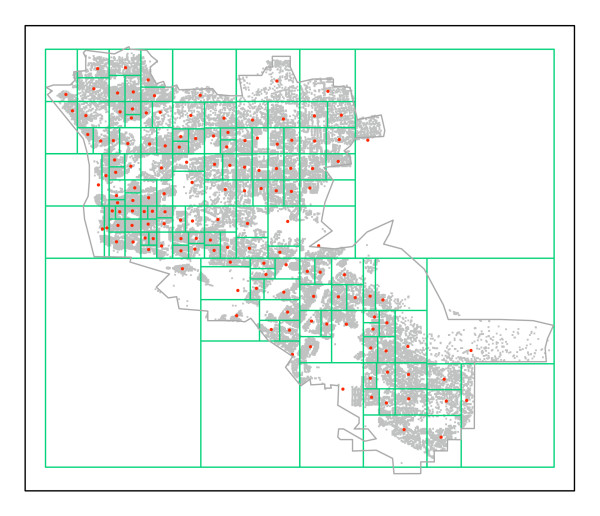
**Set 1 Epiunt areal units, with maximum 1,000 births, for RC B**. Population centroids for each unit are red. All located births are gray points.

Using the Epiunit NHalf algorithm, Set 1, n_max_= 1,000, and Set 2, n_max_= 2,000, areal units were created for each RC. Summary descriptions are in Table 1. The Set 1 partitions determined for the two RCs are illustrated in Figures 6 and 7.

**Table 1:** Summary population statistics for Set 1 and Set 2 aggregation units.

Study Region	Units of analysis	Unit population		
		Minimum	Median	Maximum
RC A	348 Set 1 areal units	334	561.5	1000
	175 Set 2 areal units	975	1117	2000
RC B	148 Set 1 areal units	486	605	1000
	74 Set 2 areal units	958	1156	2000

### Spatial test results

**Figure 8:**
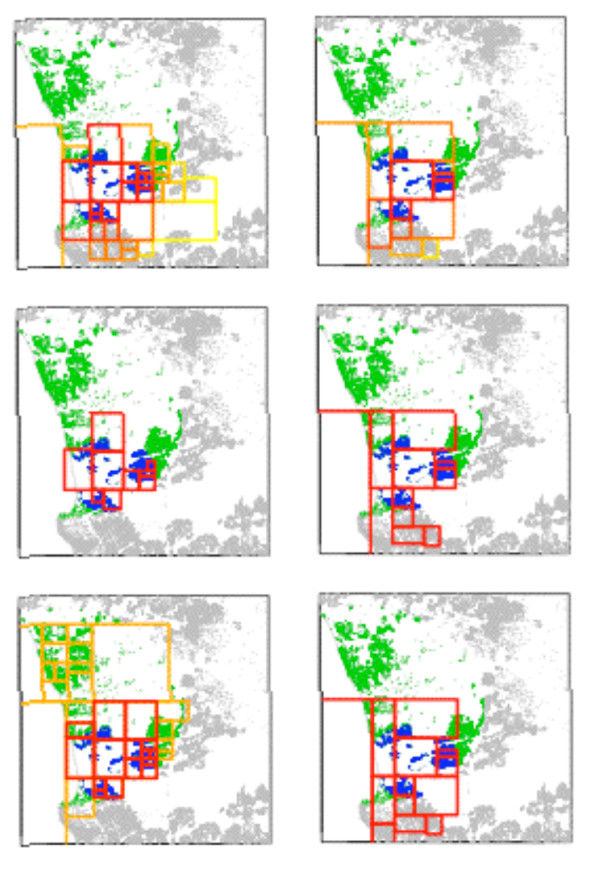
**RC A: Qualifying LACT results**. Rows, from the top, represent the LACTs (Episcan, FleXScan, and SaTScan areal) and columns, from the left, represent the areal unit sets (Set 1 (maximum unit pop. = 1000), Set 2 (maximum unit pop. = 2000). The underlying green and blue points indicate the qualifying SaTScan point clusters. All other located births are gray points. On Row 1, the Episcan display uses a heat map to indicate the most likely cluster area within reported significant clusters. In Row 3, Column1, SaTScan areal Set 1 had two overlapping significant clusters, both shown here in different colors.

**Figure 9:**
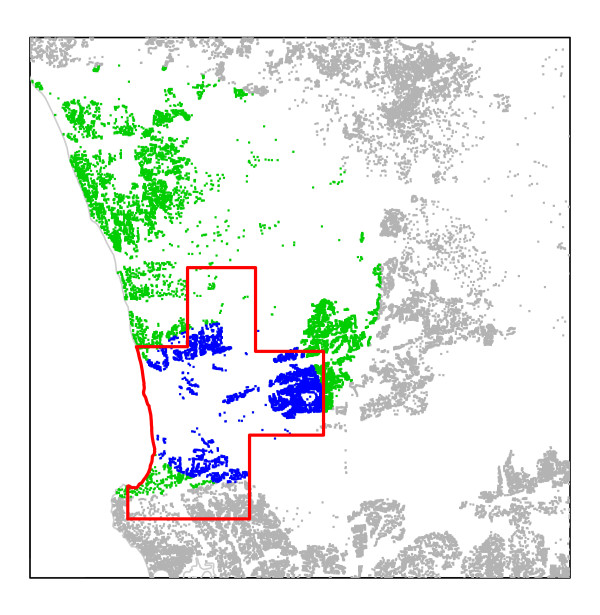
**Consensus Cluster for RC A outlined in red**. The qualifying SaTScan point clusters are green and blue; all other located births are gray.

The results of all spatial tests for both study regions are summarized in Tables 2, 3 and 4. The qualifying results, according to the testing procedure detailed below in the Methods section, are also mapped in Figures 8 and 9. All secondary clusters of FleXScan were highly non-significant and are not shown.

**Table 2:** Results of SaTScan tests that allow overlap among identified statistically significant clusters.

Study Region	Units of analysis	Highly significant clusters, p ≤ 0.01		Significant clusters, 0.01 < p ≤ 0.05		Maximum Relative Risk	Accepted contributing clusters
		
		Count	Population range	Count	Population		
		
RC A	Set 1^1^	3	8919, 25863	0	n.a.	2.27	2
	Set 2^2^	2	16390, 27967	0	n.a.	1.91	1
	Points^3^	3	5, 7569	1	19963	2.74	2
RC B	Set 1^1^	0	n.a.	1	14197	1.48	0
	Set 2^2^	0	n.a.	0	n.a.	n.a.	0
	Points^3^	0	n.a.	1	9	63.66	0

^1 ^Maximum unit population of 1,000.

^2 ^Maximum unit population of 2,000.

^3 ^Point analysis is only done with SaTScan

Accepted contributing clusters have a p ≤ 0.05, RR ≥ 1.7 and birth population ≥ 1000.

**Table 3:** Results of Episcan tests that allow overlap among identified statistically significant clusters.

Study Region	Units of analysis	Highly significant clusters, p ≤ 0.01		Significant clusters, 0.01 < p ≤ 0.05		Maximum Relative Risk	Accepted contributing clusters
		
		Count	Population range	Count	Population range		
		
RC A	Set 1^1^	183	2914, 33009	810	1496, 33463	2.22	269
	Set 2^2^	50	9074, 32919	186	3729, 33661	1.96	126
RC B	Set 1^1^	0	n.a.	2	11775, 13697	1.50	0
	Set 2^2^	0	n.a.	1	15599	1.44	0

^1 ^Maximum unit population of 1,000.

^2 ^Maximum unit population of 2,000

Accepted contributing clusters have a p ≤ 0.05, RR ≥ 1.7 and birth population ≥ 1000.

**Table 4:** Results of MEET global clustering test and the primary cluster reported by FleXScan.

		MEET	FleXScan Primary Cluster			
			
Study Region	Units of analysis	adj p value	p value	Population	Relative Risk	Accepted contributing cluster
RC A	Set 1^1^	0.264	0.001	5357	2.94	Yes
	Set 2^2^	0.292	0.001	12814	2.15	Yes
RC B	Set 1^1^	0.181	0.037	6680	1.80	Yes
	Set 2^2^	0.172	0.006	10498	1.69	No

^1 ^Maximum unit population of 1,000.

^2 ^Maximum unit population of 2,000

FleXScan does not allow overlap among identified clusters. Accepted contributing clusters have a p ≤ 0.05, RR ≥ 1.7 and birth population ≥ 1000.

### RC A

#### Point data test results for RC A

In RC A, there were four statistically significant (p ≤ 0.05) point clusters reported by SaTScan. Two of the three highly significant (p < 0.01) clusters had fewer than ten births each and contained the same four cases. They were not statistically significant when expanded to include one additional case and were not located near the larger clusters. The remaining two large overlapping clusters had 56 cases in 7,569 births and 99 cases in 19,963 births; they formed the basis of a Consensus Cluster as defined earlier.

#### Areal data test results for RC A

SaTScan areal test confirmed the significant large cluster identified by the point test, identifying two qualifying highly significant clusters, one each in Set 1 and Set 2.

Episcan generated 1,000 clusters in Set 1 and 236 in Set 2 with p ≤ 0.05. Of these, 269 of Set 1 and 126 of Set 2 clusters had RR ≥ 1.7 and qualified for consideration. In Set 1, areal units occurring in at least 54 (20 percent of 269) of the qualifying clusters are mapped in Figure 8. For Set 2, areal units in more than 26 qualifying clusters are mapped. FleXScan's primary cluster also covers this area with p = 0.001 for both Set 1 and 2. With results from all seven LACT applications passing our criteria, RC A has a Consensus Cluster.

MEET did not detect overall clustering, p ≥ 0.264. In the face of strong LACT evidence of a single, very significant cluster, we interpreted MEET results as support for the existence of no more than one large Consensus Cluster in the study region (Figure 9).

### RC B

#### Point data test results for RC B

In RC B, the only significant point cluster identified consisted of four cases in nine births. The next most statistically significant clusters were eight cases in 112 births (p = 0.141), covering the significant cluster, and eight cases in 111 births (p = 0.136).

#### Areal data test results for RC B

**Figure 10:**
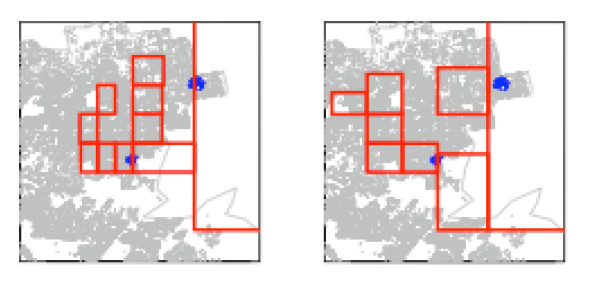
**RC B: FleXScan results, including the only qualifying result**. The only LACT result that met qualifications was FleXScan for Set 1. From the left, Set 1 and Set 2 (RR = 1.69) are outlined in red. These clusters connect the only point clusters, in blue, that had p values below 0.2 (births < 115). None of the point-identified clusters met acceptance criteria. All other located births are gray points. There is no consensus or potential cluster for RC B.

Of the 100 areal unit-based clusters that Episcan reported for each of Set 1 and Set 2, only 2 Set 1 and 1 Set 2 clusters were significant and all RRs were less than 1.7. The single significant SaTScan areal-identified cluster failed to have an adequate RR. Only FleXScan's Set 1 primary cluster qualified for consideration for a Consensus Cluster, though the RR of the FleXScan primary cluster for Set 2 was close at 1.69 (Figure 10).

The non-significant results from MEET do not support the existence of global clustering. According to this multiple test procedure, the amount of clustering in this study region was not significantly different from that expected by random.

The size of the dataset SaTScan version 7.0 can analyze is limited by available computer resources. Performing SaTScan on large sets of data points took up to five days per test on Dual 2.4 GHz single-core AMD Opteron 250 Series processors, with eight GB of memory running SUSE Linux 2.6.11.4.

## Discussion

From the simulations, it appears that LACTs do not perform well for a true RR below 1.7. In the second simulation set, the median SE and p values improve dramatically at this point. In the first simulation set, reduced median SE and SP occurred together for RR = 1.5 compared to higher RR levels in all but two of the 32 different areal unit size and cluster shape scenarios. Balancing the increasing median SE and decreasing median SP that occur with increasing unit size, we decided on n_max _of 1,000 and 2,000 for testing units in our procedure.

The second set of simulations informed the procedure's cutoffs for observed RRs and p values in the LACTs. The unsettling observation from Figure 4 that the median observed RR is at least 2.0 when the distribution is random (RR = 1.0) in all scenarios demonstrates the limitations of tests with SP of 0.98 when analyzing rare occurrences. For the high observed RR below a true 1.7, we must rely on a maximum p value limit and requiring multiple test agreement to limit these false positives. But as the median observed RR does estimate the median true RR well starting at 1.7, we feel that requiring a minimum observed RR of 1.7 improves the test validity. We did not choose a higher cutoff because the lower limit of the 90 percent range for the circular scan on the rectangular cluster was below 2.0 until the true RR was 4.0.

Since the overestimated observed RRs below true RRs of 1.7 coincide with high median p values, loosening the usual significance limit of 0.05 would bring more of these false positive results into our analysis. At true RR = 1.7, all p values but the circular scan for rectangular clusters were below 0.05. Any lower limit would not detect this level of clustering. Further, if using multiple tests, requiring a p value of 0.05 from each LACT results in a lower overall p value. We therefore kept the significance cutoff for all tests at 0.05.

Incorporating these criteria in our procedure, as we saw in RC B, it characterizes a region as having no clustering although one areal LACT result had a qualifying result. The significant point-based cluster had only four cases and the global test was non-significant. The conclusion of no clustering seems a reasonable inference since the only qualifying result (FleXScan) connected the two small point-based clusters (p = 1.4) with eight cases each. Based on the simulations, it is more likely that the FleXScan result is spurious than that there is a cluster here. In RC A, MEET was also not significant, but a Consensus Cluster was determined nonetheless. As Kulldorff et al [1] noted, global tests, including MEET, may not detect clustering if it consists of only one or two clusters within the entire study area.

For this low incidence disorder, our results for SaTScan agreed with the findings of Gregorio et al[18], who compared the results from SaTScan on data both in point form and aggregated by census units. We both found general agreement for cluster detection between the point data and their lowest level of census data aggregation and our smaller areal units. On the other hand, Episcan and FleXScan, using the areal units, sometimes indicated smaller clusters than those indicated by SaTScan with either point or aggregated areal data. Individual data are generally presumed to be more accurate than aggregated data. Perhaps for very low incidence diseases, small, population-defined areal units, such as Epiunts, are more useful in spatial tests such as SaTScan and Episcan. Comparing the accuracy of these two test input forms for a very low incidence disease would be an interesting simulation study.

As both SaTScan and FleXScan are available with graphical user interfaces, they are more likely to be used than the less-user friendly Episcan and MEET. This is unfortunate as Episcan yields more spatial information than the other LACTs, as was shown in Figure 8, and it is an integral part of this procedure. Episcan probably has higher SE than our simulations demonstrated; it incorporates more than just the most likely cluster in its result.

## Conclusion

This procedure is designed to minimize false positive results without compromising power unnecessarily. For an exploratory spatial analysis, a false positive can be a false alarm, diverting research efforts and funds by generating false hypotheses from the many possible environmental risk factors to be assessed in any identified area. Different situations may warrant different procedures. Our simulations looked at the observed and known RRs for a variety of cluster shapes. The overestimation of the low (1.0 – 1.7) true RRs cautions against accepting higher p values to compensate for low power. Limiting the p values to 0.05 for significance will remove much of these exaggerated results. Results from a cluster larger than the five percent one we simulated could have a narrower 90 percent range, though the average estimate might not change.

We were surprised to see the magnitude of the downward bias of observed RRs for true RRs over 3.0, especially for circular scans with the rectangular clusters. Variable elliptical scans showed less of a downward bias meaning Episcan would therefore be the more reliable test for unknown cluster shapes. This does warrant a caveat for inferences from LACTs. The RR from a LACT will be lower for true RRs over 3.0 when compared to another association obtained from a different statistic.

To our knowledge, this is the first report combining multiple spatial tests into a systematic approach for a large cohort of individual locations. In an area like California, with county population densities ranging from 2 to 8,714 per square mile [19], only a cohort study provides accurate spatial distributions. While any spatial analysis of events with prevalence less than 0.005 remains challenging, for truly exploratory research, as our simulations demonstrate, this procedure is an improvement over the common practice of using one of the available LACTs and a global test with no limits on acceptable RR. By requiring confirmation from multiple tests and setting RR and p value limits for LACTs, we expect to reduce the percentage of false positive results. By considering Potential Clusters identified in only one set of partitioning areal units, we expect to reduce the false negative results due to aggregation units.

Our simulations illustrated median SE < 0.8 for rectangular clusters with true RR < 3.0. But Figure 3 shows that one-third of simulations are RR = 2.0 had SE ≥ 8.0. The lower SE values are associated with the more elongated clusters. For these shapes, Episcan and FleXScan may be more powerful than the single maximum likelihood cluster. In use, the three different LACTs showed a surprising agreement on an area for a cluster with a significant p value and a RR over 2.0 in almost half of the 21 DDS RC catchment areas studied. Since these clusters easily pass our criteria using parallel multiple tests, we feel we have not been too stringent. This procedure identified 10 Consensus Clusters and 2 Potential Clusters within the 21 RC areas.

Further validation for this procedure came from demographic analysis of the Consensus Clusters. In multiple regressions using individual demographic data on known risk factors, almost all of the defined cluster populations showed strong, significant associations with one of these variables, indicating actual clusters. In such clusters that appear to be explained mainly by demographics, either environmental risks occur in a non-geographical distribution, i.e. in the home or workplace, or there is some risk attached to demographic status. If so, then environmental exposure research is likely to be more fruitful if focused on the home and workplace. If not, then we can search for geographically distributed environmental risks within the remaining unexplained clusters.

## Methods

### Simulations of the study questions

Simulations were based on the geocoded maternal address at birth for all births during a period of five years in a two county area of California, USA, with 219,417 locations in a land area of 8,375 square miles. The background prevalence was chosen as 0.004, which was the overall background incidence in our statewide study dataset. Each simulation had one randomly located cluster containing approximately five percent of the locations. For each simulation, Bernoulli experiments were performed at all locations with the risk given by the scenarios below.

For every scenario, two sets of 100 simulations each were performed. One set used a circular shape for the cluster; the second set used a rectangular shape with aspect ratio varying from 2 to 10, simulating elongated clusters, and a random orientation.

For all simulations, we used the Episcan procedure to produce the most likely cluster for each simulation using (a) only circular (representing SaTScan) and (b) elliptical cluster scan shapes.

Scenario 1. To explore the question of the effect of unit population size, assess all combinations of:

a. RR of 1.5, 2.5, and 5.0

b. n_max _of 500, 1000, 2000, and 4000

Scenario 2. To explore the question of the effect of the true cluster RR on the LACT results, the unit n_max _was fixed at 1000 and the RR of the actual cluster was varied from 1.0 (random distribution) to 5.0 in steps of 0.1. Simulations and all additional programming were done in R version 2.n [20].

### Spatial tests

SaTScan uses a specified shape, usually a circle, to create a series of clusters with each data point or every areal unit's centroid as a starting point, assigning the most likely cluster's empirical p values based on 999 or 9,999 Monte Carlo randomized case distribution simulations over the study population distribution [16]. The test is based on the binomial distribution for individual data points and the Poisson distribution for data in areal units of aggregated populations. FleXScan [10] and Episcan [11], build upon this method to highlight clusters of indeterminate shape. Episcan uses the binomial probability distribution on either the centroids of aggregated areal units or disaggregated point data [11], while FleXScan uses the Poisson probability distribution on aggregated areal units [10]. Since we are concerned with rare events with prevalence under one percent, the two distributions produce a similar model for testing clusters.

FleXScan takes a nearest-neighbor approach to building clusters so it is free to connect up to a prespecified maximum number of areal units with elevated prevalence in indeterminately-shaped non-overlapping clusters [10]. Episcan generates sets of elliptical clusters of varying sizes for every data point using each of its neighboring data points as the second elliptical focus [11]. Episcan reports a prespecified number of the most significant clusters, usually at least 1,000. Using these clusters, the areal units occurring in the largest number of, often overlapping, clusters with p values below a specified maximum are highlighted rather than a single most likely cluster. In this way, Episcan is not limited to its elliptical cluster design. Both FleXScan and Episcan can also be restricted to the same set of circular clusters used in SaTScan as a special case. Episcan always includes the circles as part of its analysis while FleXScan requires a separate analysis.

MEET is a global clustering test that searches for the most likely cluster size for the areal data being studied by varying a scale parameter, λ, over a default range. It then tests the hypothesis of no clustering of cases given the background population distribution versus the alternate hypothesis that there is some clustering [14]. It is most powerful in detecting multiple clusters of similar size. A single adjusted overall p value is reported for the computed clustering statistic, C, along with the distribution of p values over the range of λ. MEET also indicates which areal units contributed the most to C. If C is statistically significant, these areal units are likely cluster candidates.

### Parameter settings for spatial tests

#### Maximum allowed size for detecting clusters

Each LACT has recommended default parameter settings. The default maximum cluster size to be detected for SaTScan is 50 percent of the locations; for Episcan it is 15 percent.

To maintain consistency among tests, a single parameter setting was used for all analyses. For Episcan and SaTScan, we found higher agreement between the point and areal tests with a maximum cluster size of 15 versus 50 percent for low incidence data and therefore used 15 percent in our procedure. Using a maximum of 15 or 20 areal units, FleXScan typically identified the same clusters; however, occasionally (< 5 percent), it identified two different clusters, anchored at the same unit, but continuing in different directions. We used the default setting of 15 percent, as we found no compelling reasons to not do so.

#### SaTScan secondary cluster reporting limit

After reporting the primary most likely cluster, SaTScan's cluster reporting setting dictates what secondary clusters, with lower p values, will be reported. The default setting of "no geographical overlap" reports only secondary clusters that share no locations with the primary cluster. Other settings allow various levels of primary cluster overlap for the secondary clusters. Using these settings for both point and areal data, we found some overlapping clusters with higher, yet still very significant p values that had a higher RR than the primary cluster. In densely populated areas, these overlapping clusters may indicate a non-circular cluster, which can be compared to those identified using the more flexible LACTs. Using any of the cluster reporting settings that were more restrictive than "no pairs of centers both in each other's clusters" SaTScan failed to detect some statistically significant overlapping clusters; however, less restrictive settings examined gave no additional useful information.

### Data format considerations

#### Epiunit areal units

The areal units used in the simulations were produced using Epiunits, the geo-aggregation tool for spatial point data developed for this study (Christiansen, unpublished), to minimize the number of units with very low population and/or zero case counts.

**Figure 11:**
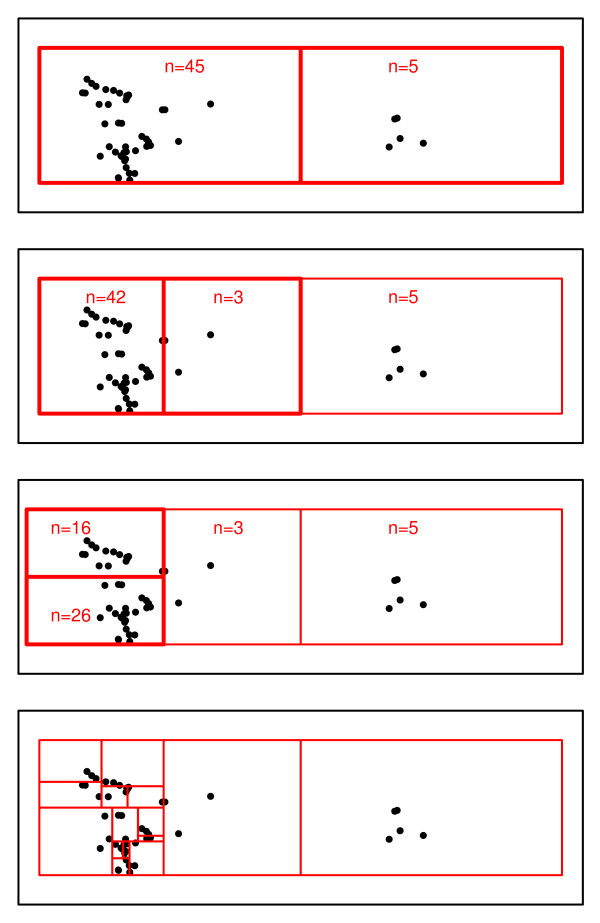
**A simplified illustration of the Epiunit NHalf areal unit partitioning algorithm with n_max _= 5**. From the top, for a study population of 50 in a rectangular study area, Step 1, a rectangle is drawn around the study area and the first partition bisects the longer axis. Step 2, any unit that contains more than n_max _= 5 locations is bisected on its longer axis. If all units created have at least 3 (> 2.5, or one-half of n_max _= 5) locations, assess whether either unit contains more than 5 locations. Step 3, bisect the longer axis of any unit containing more than 5 units. Step 4, if a unit has less than 2.5 locations go back and make a partition into two units such that the location count is approximately equal. Repeat steps 3 and 4 recursively until all units have between 3 and 5 locations.

The Epiunit NHalf algorithm creates areal units (Epiunits) by first creating a rectangle to encompass the study region. This rectangle is then subdivided by bisecting its longer edge and subsequently created rectangles will be similarly bisected until the size of the population in a rectangle is no more than twice the user-specified maximum areal unit population, n_max_. Once the population in either rectangle created by the next bisection is less than one-half n_max_, the final 'bisection' of each rectangular area is created by moving the bisecting line until its population, not its area, is halved to create the actual Epiunits. In this way, only when Epiunits have multiple points on the final dividing line, one of them may have a population less than half of the maximum specified. An advantage of this method is that the Set 1 units are not precisely nested within the Set 2 units so that clusters crossing areal unit boundaries will not be divided exactly the same way by both sets of areal units. A simplified example of the algorithm is shown in Figure 11.

By specifying a maximum population per areal unit, the user effectively sets the minimum unit population at one-half of n_max_. This creates a set of areal units with more homogeneous populations without restricting the range of geographical sizes, which can be large.

For this procedure, two sets of areal units were created for testing by using the Epiunit NHalf algorithm with n_max _set at either 1,000 (Set 1) or 2,000 (Set 2). If a study region population density is less than two per square mile, fewer than 30 areal units are generated for Set 2, or 2,000 is more than the maximum population for the Episcan or SaTScan scanning window, a Set 3 with n_max _of 500 should replace Set 2 in the analysis.

#### Point data

SaTScan is the only LACT that can currently analyze a large population without aggregating point locations into areal units. With low prevalence, smaller clusters are very sensitive to the change of one or two cases. Logically, the lower the prevalence, the larger the cluster population needed for a stable result.

We found SaTScan could identify highly significant point-based clusters, with an extremely high RR, that were composed of two cases in less than 50 births over five years. However, these clusters were not contained within larger clusters of raised prevalence. Particularly with a rare event with a genetic component, two people in one location should not be regarded as a cluster if there are no other events in the surrounding group of some specified minimum size.

We chose to require a minimum cluster size of 1,000 population as a very liberal limit since areas we analyzed separately had a range of overall incidence of 0.0020 to 0.0088.

### Test procedure of parallel tests and their parameter settings

SaTScan, Episcan, FleXScan and MEET were applied to two different sets of Epiunit-created areal units. SaTScan was also applied to the disaggregated point data. Together they gave a more accurate assessment of possible geographic clustering of a low incidence disease. The following settings were used:

#### SaTScan

For both areal units and point locations, SaTScan identified high risk clusters only, with maximum cluster size of 15 percent of the study population. The cluster reporting parameter setting was "no pairs of centers both in each other's clusters". Point location clusters were assessed with the binomial distribution and areal unit clusters with the Poisson distribution.

#### Episcan

Episcan was run with maximum cluster size of 15 percent of the locations and maximum of 10 percent of other areal units used as a second focus for each initial point. The number of clusters to be created and evaluated was the lower of 1,000 or the maximum possible for the set of areal units. Episcan displays the areal units occurring in more than a designated percentage, here 20, of the qualifying clusters with heat colors indicating their frequency.

#### FleXScan

FleXScan was run with the default settings, including maximum cluster size of 15 areal units. FleXScan reports only non-overlapping clusters, usually producing one or two clusters with the lowest p value of any LACT and statistically very non-significant secondary clusters.

The RR that is included in the FleXScan output uses the incidence in the entire study region as the denominator and so was recalculated with the incidence in the study region outside of the cluster as the denominator to conform to RR measures produced by the other LACTs.

#### MEET

MEET was run with latitudes adjusted by cos(mean(degrees longitude)*pi/180) instead of a fixed number in the distance measure, d_ij_, when computing neighbor distances, and the default one to twenty range for λ, the scale parameter.

### Defining Consensus Clusters of high incidence

We defined a Consensus Cluster as occurring when the point LACT identified a cluster of at least 1000 births and all three areal LACTs on both sets of areal units indicated this same area. All seven LACT results must meet criteria of p ≤ 0.05 and RR ≥ 1.70. Therefore, if five cases per thousand are expected, the minimal qualifying point cluster would contain nine cases. The results of this composite test procedure, having passed multiple tests and rules, were referred to as Consensus Clusters to distinguish them from the statistically significant clusters defined by the individual tests. A Consensus Cluster did not require MEET to be significant. Such a result implied that there was no significant clustering in the remainder of the study area outside the defined cluster.

The actual cluster estimate was defined as the total of the areal units in the FleXScan cluster and areal units occurring in more than 75 percent of qualifying Episcan-defined clusters approximately covering the same area from either Set 1 or 2, whichever cluster estimate is smaller. There could be more than one non-overlapping cluster so defined in a study region.

### Defining Potential Clusters less stringently

As noted before, a cluster could be masked by the boundaries of one set of partitioning areal units and not another. So we defined a Potential Cluster as an area that qualified as a Consensus Cluster in the results of the point test and only Set 1 or Set 2. The defined area is as above.

### Assessing non-environmental contribution to the defined clusters

If there are known demographic risk factors for the disorder that could cluster geographically, i.e. socioeconomic status, their contribution to the Consensus Clusters must be explored. For areal units analyzed using the Poisson distribution, both SaTScan and MEET can evaluate categorical population covariates by stratification within the units. With a rare disorder, this is not feasible for more than one or two variables because the stratification would lead to most strata having no cases. Instead, logistic or Poisson regression should be performed, using individual level demographic information comparing locations within the cluster with those outside, regardless of case status. The strength and size of these associations will affect the likelihood that the cluster has a geographically distributed environmental association.

## Competing interests

The authors declare that they have no competing interests.

## Authors' contributions

KVM carried out the spatial analysis procedure development in concert with LEC, performed all spatial analysis and drafted the manuscript.

LEC developed custom software, performed all simulations and made refinements on existing software working with KVM. He revised the manuscript critically for important intellectual content.

IHP, RA and TEC all participated in the study design and all decisions on the final procedure parameters. They all revised the manuscript critically for important intellectual content.

All authors read and approved the final manuscript.
